# Prehospital respiratory interventions during six waves of COVID-19: results from Israel’s Emergency Medical Services system

**DOI:** 10.1186/s12873-025-01279-9

**Published:** 2025-07-06

**Authors:** Maximilian P. Nerlander, Evan Avraham Alpert, Roman Sonkin, Ziv Dadon, Ari M. Lipsky, Eli Jaffe

**Affiliations:** 1 Center for Disaster Medicine and Traumatology, Linköping University, Linköping, Sweden; 2 Community Division, Magen David Adom, Or Yehuda, Israel; 3 Faculty of Medicine, Hebrew University of Jerusalem, Jerusalem, Israel; 4 Department of Emergency Medicine, Hadassah Medical Center, Ein Kerem, Jerusalem, Israel; 5 Department of Internal Medicine, Soroka Medical Center, Beer-Sheva, Israel; 6 Jesselson Integrated Heart Center, Shaare Zedek Medical Center, Eisenberg R&D authority, Jerusalem, Israel; 7 Department of Emergency Medicine, HaEmek Medical Center, Afula, Israel; 8 Rappaport Faculty of Medicine, Technion-Israel Institute of Technology, Haifa, Israel; 9 Faculty of Health Sciences, Ben-Gurion University of the Negev, Beer-Sheva, Israel

**Keywords:** SARS-CoV-2, COVID-19, Emergency Medical Services, Prehospital, Interventions, Covid, Waves, Vaccines

## Abstract

**Background:**

Despite COVID-19 having been the subject of extensive scientific research, there is a paucity of studies on the respiratory management needs of patients in the pre-hospital setting. This retrospective cohort study utilizes data from Magen David Adom (MDA), Israel’s Emergency Medical Services (EMS) to investigate how prehospital respiratory management needs changed throughout the first six waves of the COVID-19 pandemic in Israel.

**Methods:**

All EMS responses due to respiratory complaints, from March 21, 2020, to July 31, 2022, were included. Odds ratios (ORs) for each wave were calculated for each intervention with the previous wave as reference. Wave 1 (W1) was compared to a pre-pandemic period.

**Results:**

The study included 141,027 responses. Throughout the pandemic, no endotracheal intubations were performed. The use of mask-based 90% FiO_2_ decreased from the pre-COVID-19 period to W1 (OR 0.61, *p* < 0.0001), increased during waves 2–3 (OR 1.24, *p* < 0.0001 [W2]; OR 1.11, *p* < 0.0001 [W3]), and plateaued throughout W5 and W6 (OR 0.99, *p* = 0.71 [W5]; OR 0.01, *p* = 0.8 [W6]). The use of nasal cannula increased throughout the six waves (OR 1.2 [W1]; OR 1.48 [W2]; 1.39 [W3]; OR 1.45 [W4]; 1.11 [W5]; 1.24 [W6], *p* < 0.05). The use of nebulized bronchodilators decreased from the pre-pandemic period to W1 (OR 0.41, *p* < 0.0001). From W3 to W6, the use increased significantly for each wave (OR 1.43 [W4]; OR 1.12 [W5]; 1.31 [W6], *p* < 0.05).

**Conclusions:**

MDA advised staff not to perform endotracheal intubations during the pandemic due to concerns of transmission through aerosol. Similarly, the initial drops in high-concentration O2 and nebulized bronchodilators may be due to concerns with its aerosolizing potential. The gradual replacement of high-concentration prehospital oxygen by mask with nasal cannulas over the pandemic waves may be due to the decreased pathogenicity of the new strains and expanded vaccination coverage. The increased use of nebulized bronchodilators seen during the latter waves may be due to the re-emergence of non-covid pathogens with greater bronchoconstrictive effect as social restrictions were eased. Further, as vaccine coverage expanded among providers over time, these may have been more comfortable administering bronchodilators.

## Background

COVID-19 is primarily a respiratory infection, and many patients, especially in the beginning of the pandemic, required some form of respiratory support. Among patients who developed severe disease, the predominance of respiratory symptoms is well documented. A literature review of 194 COVID-19-related articles identified that cough, dyspnea and pulmonary infiltrates were the most common presentations of severe illness [1]. A study from Wuhan, China, in the early phase of the pandemic, found that dyspnea was reported in 63.9% of patients with COVID-19 who were admitted to the Intensive Care Unit [2]. Further, a New York City-based prospective cohort study involving 1150 patients with COVID-19 found that out of 257 patients who developed severe disease, 79% required mechanical ventilation [3]. 

Despite COVID-19 having been the subject of extensive scientific research, including in the prehospital setting, there is a paucity of studies on how the respiratory management needs of patients are reflected in the prehospital setting and how these have changed throughout the different waves of the COVID-19 pandemic [4, 5]. These are likely affected by several factors: First, the emergence of new strains has affected the pathogenicity of the virus. As new variants of COVID-19 have appeared, Case Fatality Rate (CFR) appears to have decreased while the attack rate has increased. The Omicron variant appears to be more infectious, but causes a milder form of disease compared to the earlier Delta variant [6–9]. However, the global shortage of COVID-19 testing kits in the beginning of the pandemic may have resulted in underdiagnosis and undercounting, thus artificially increasing the CFR of the early strains. Second, the expansion of vaccine coverage decreased the likelihood of severe disease and therefore likely affected respiratory management needs in the prehospital setting as well [9–12]. Third, the emergence of non-COVID-19 respiratory pathogens as social restrictions were lifted may have affected the clinical needs of patients [13–18]. Lastly, changing clinical practices as the virus was better understood will also have been reflected in the prehospital setting. Initial concerns about aerosolizing respiratory interventions increasing the risk of infection to clinicians may not be as risky as first thought, and, as the virus is better understood, the practice has changed to focus more on patient needs [19–23]. 

Magen David Adom (MDA) is Israel’s Emergency Medical Services (EMS) System and accounted for the majority of the prehospital care provided during the COVID-19 pandemic. MDA, through its comprehensive nationwide data collection on EMS responses, provides an opportunity to address the paucity of studies of prehospital respiratory management needs and to understand these at a national level. Thus, the objective of this study was to utilize national data from MDA’s Command and Control database to evaluate population-wide prehospital respiratory management needs throughout the first six waves of the COVID-19 pandemic in Israel.

## Methods

This is a retrospective cohort study utilizing routinely collected anonymized data from MDA’s Command and Control database. All EMS responses due to respiratory symptoms in Israel, from March 21, 2020, to July 31, 2022, were included. These represented unique EMS responses and not individual patients. EMS responses where the primary complaint was not coded as respiratory-related were excluded. Confirming the diagnosis of COVID-19 in the prehospital setting was not possible; further, electronic patient records in Israeli hospitals cannot generally be matched to MDA data and does therefore not allow for retrospective data entry once the diagnosis of COVID-19 has been confirmed. Variables included response date, demographic data and respiratory interventions including endotracheal tube (ETT), laryngeal mask airway (LMA), continuous positive airway pressure (CPAP), bag valve mask (BVM), oxygen mask (90% FiO_2_), oxygen mask (60% FiO_2_), nasal cannula, and nebulized bronchodilators.

Changes in respiratory management needs throughout the pandemic were analyzed based on the first six waves of COVID-19 in Israel. National data from the Israeli Ministry of Health were used to determine the duration of each wave. While there is no scientific consensus on the start and end dates of a COVID-19 wave, for this study, the timeframes of a wave were defined as a margin of 12% of the sum of the peak height of the wave, minus the baseline between waves. The beginning of the wave was calculated relative to the preceding baseline and to the end of the next baseline. Additionally, to provide a reference period for Wave 1 (W1), a corresponding calendar period in 2019 was selected to account for temperature-related factors in transmissibility of respiratory viruses. Table 1 details the time intervals used for the analysis, and the dominant strains of COVID-19 in each period:

**Table 1:** Reference period, waves, and dominant strains

Period	Dates	Dominant Strain in Israel
Reference	March 21, 2020, to May 5, 2019	N/A
Wave 1	March 21, 2020, to May 5, 2020	Original strain
Wave 2	July 3, 2020, to October 30, 2020	Original strain
Wave 3	November 30, 2020, to March 23, 2021	Alpha B.1.1.7
Wave 4	July 26, 2021, to October 20, 2021	Delta
Wave 5	January 8, 2022, to April 3, 2022	Omicron
Wave 6	May 15, 2022, to July 31, 2022	Omicron

The proportions of prehospital respiratory interventions during each wave were compared to those of the preceding wave and Odds Ratios (OR) calculated. Additionally, a separate variable was created, defined as having undergone at least one of the interventions included in the data, and analyzed as above. Data were extracted to Microsoft Excel (Microsoft 108 Excel, Version 16.33, Seattle, WA, USA), and cleaned and analyzed using JMP 13 (100 SAS Campus Drive, Cary, NC, USA). This study was approved by the Scientific Committee of MDA and the Helsinki Committee of the Shaare Zedek Medical Center (study number 0145-21-SZMC) with a waiver of informed consent.

## Results

The final analysis included a total of 141,027 responses. Table 2 details the demographic characteristics of the study population. The gender distribution was even, with 48.8% of patients being male. The median age was 71.4 years [IQR: 52.7–84.0] (Table 2). A total of 9,002 (6.38%) of responses were in children. Across the study period, most responses involved the administration of a respiratory intervention (57.0%). The most commonly employed intervention was mask-based 90% FiO_2_ (36.6%), followed by nasal cannula (13.3%), and nebulized bronchodilators (8.4%). The use of mask-based 60% FiO_2_, bag valve mask, CPAP, LMA, and ETT each accounted for less than 3% of the total cohort.

**Table 2:** Characteristics of the study population, *n* = 141,027

	Reference	W1	W2	W3	W4	W5	W6	Total
Dominant Stain of Covid-19	N/A	Original strain	Original strain	Alpha B.1.1.7	Delta	Omicron	Omicron	N/A
Age (Median [IQR])	76.1 [62–85.3]	66.2 [44.1–81.3]	68.1 [48.8–82]	70 [50.1–83]	68.7 [47.3–82.4]	74.2 [58.1–85.2]	75.1 [61.1–85.2]	71.4 [52.7–84.0]
Male (n, %)	3,383 (48.49%)	4,163 (51.12%)	8,913 (49.81%)	10,675 (48.79%)	7,380 (48.43%)	8,760 (47.42%)	5,728 (48.97%)	72,141 (48.80%)
Female (n, %)	3,594 (51.51%)	3,981 (48.88%)	8,980 (50.19%)	11,206 (51.21%)	7,860 (51.57%)	9,713 (52.58%)	5,968 (51.03%)	75,863 (51.20%)
Interventions								
Any (n, %)	4,520 (5.32%)	3,695 (4.35%)	9,243 (10.88%)	12,228 (14.39%)	8,355 (9.83%)	10,587 (12.46%)	7,124 (8.38%)	80,452 (57.0)
Mask 90% FiO2 (n, %)	3,107 (5.68%)	2,666 (4.88%)	6,740 (12.33%)	8,759 (16.02%)	5,202 (9.51%)	6,270 (11.47%)	3,986 (7.29%)	51,578 (36.6)
Nasal cannula (n, %)	330 (1.73%)	459 (2.40%)	1,457 (7.63%)	2,405 (12.60%)	2,316 (12.13%)	3,058 (16.02%)	2,302 (12.06%)	18,758 (13.3)
Nebulized Bronchodilators (n, %)	971 (7.58%)	509 (3.98%)	1,009 (7.88%)	1,157 (9.04%)	1,123 (8.77%)	1,508 (11.78%)	1,222 (9.54%)	11,834 (8.4)
Mask 60% FiO2 (n, %)	367 (9.16%)	210 (5.24%)	465 (11.61%)	555 (13.85%)	313 (7.81%)	412 (10.28%)	237 (5.92%)	3,639 (2.6)
Bag valve mask (n, %)	132 (3.80%)	123 (3.54%)	447 (12.88%)	457 (13.17%)	339 (9.77%)	430 (12.39%)	327 (9.42%)	3,338 (2.4)
Continuous Positive Airway Pressure (CPAP) (n, %)	148 (8.98%)	9 (0.55%)	91 (5.52%)	177 (10.74%)	145 (8.80%)	230 (13.96%)	169 (10.25%)	1,500 (1.1)
Endotracheal Tube Intubation (ETT) (n, %)	107 (100.00%)	0 (0.00%)	0 (0.00%)	0 (0.00%)	0 (0.00%)	0 (0.00%)	0 (0.00%)	0 (0.0)
Laryngeal Mask Airway (LMA) (n, %)	10 (100.00%)	0 (0.00%)	0 (0.00%)	0 (0.00%)	0 (0.00%)	0 (0.00%)	0 (0.00%)	0 (0.0)

**Fig. 1:**
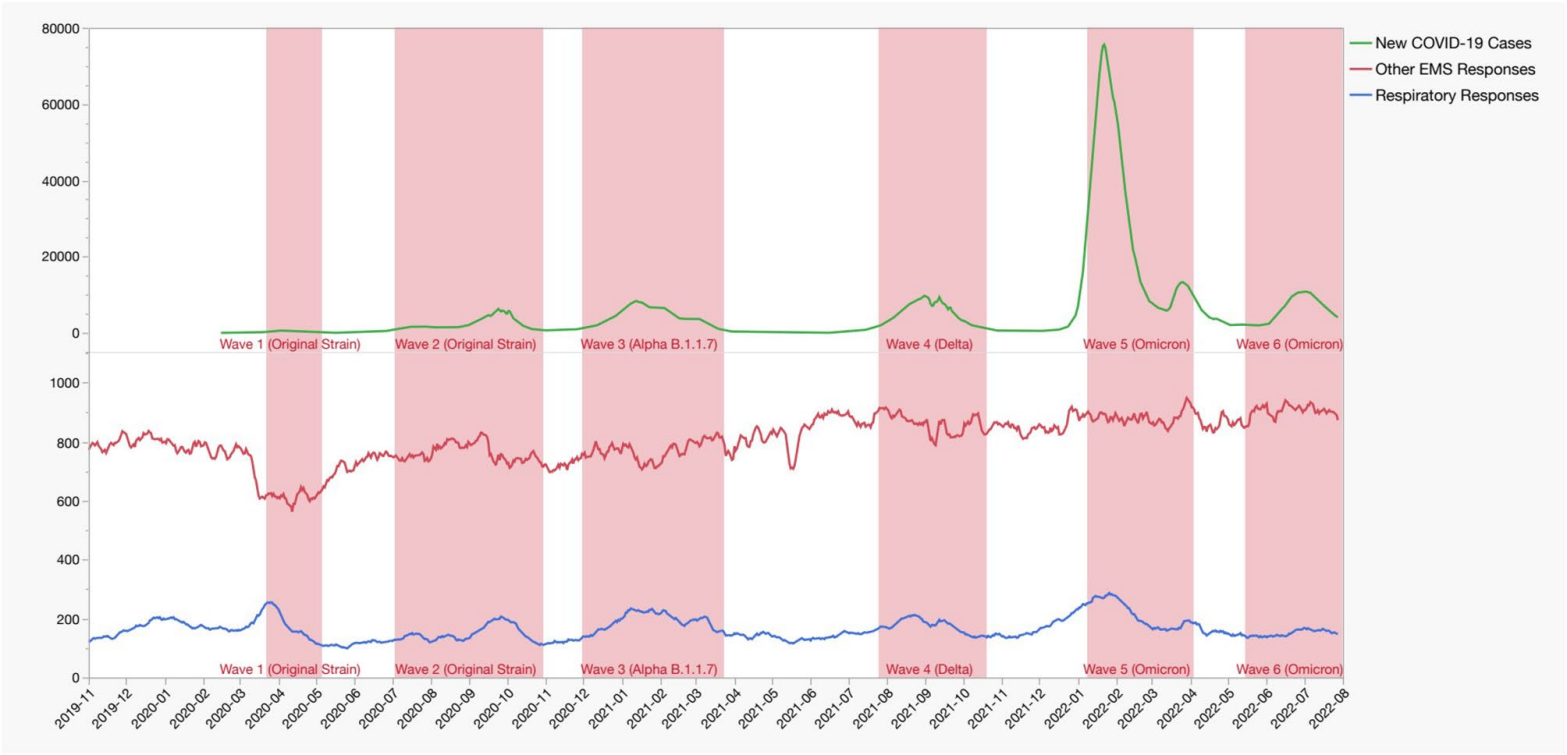
Daily number of new confirmed COVID-19 cases in Israel, respiratory responses, and other EMS responses, 7-day moving average

Figure 1 details the total daily respiratory responses across the study period together with the daily number of new confirmed COVID-19 cases in Israel and daily non-respiratory related EMS responses. The daily number of respiratory-related EMS responses peaked during W1-5, corresponding to peaks in new daily positive COVID-19 cases. However, during W6, there was no corresponding increase in respiratory-related EMS responses. By contrast, non-respiratory EMS responses decreased during W1 and then remained constant throughout the study period.

Table 3 details the results of the comparative analysis of respiratory interventions between the waves. The utilization of mask 60% FiO_2_, BVM, CPAP, ETT intubation and LMA was not included in the comparative analysis given very limited use, being administered to less than 3% of patients or not at all. The odds of any prehospital respiratory intervention being performed decreased between the corresponding pre-COVID-19 period and W1 (OR 0.45, *p* < 0.0001). Throughout W2-3 and 5–6 the odds increased (OR 1.29, *p* < 0.0001 [W2]; OR 1.19, *p* < 0.0001 [W3]; OR 1.11, *p* < 0.0001 [W5]; 1.16, *p* < 0.0001 [W6]). The odds for the use of mask-based 90% FiO_2_ decreased from the pre-COVID-19 period to W1 (OR 0.61, *p* < 0.0001) and increased during waves 2–3 (OR 1.24, *p* < 0.0001 [W2]; OR 1.11, *p* < 0.0001 [W3]). During W4, these odds decreased (OR 0.78, *p* < 0.0001) and then plateaued through W5 and W6 (OR 0.99, *p* = 0.71 [W5]; OR 1.01, *p* = 0.8 [W6]). The odds for nasal cannula use increased throughout the six waves (OR 1.2 [W1]; OR 1.48 [W2]; 1.39 [W3]; OR 1.45 [W4]; 1.11 [W5]; 1.24 [W6], *p* < 0.05 [consecutive wave pairs]). Between the pre-COVID-19 period and W1, the odds decreased for the use of nebulized bronchodilators (OR 0.41, *p* < 0.0001). There was no significant difference in these odds during W2-W3 (OR 0.90, *p* = 0.05 [W2]; OR 0.93, *p* = 0.13 [W3]). Throughout W4-6, the odds increased significantly for each wave (OR 1.43 [W4]; OR 1.12 [W5]; 1.31 [W6], *p* < 0.05) [24–29].

**Table 3:** Odds ratios for respiratory interventions across the six waves. Each wave was compared to the previous one, W1 was compared to pre-COVID-19. (*n* = 141,027; pre-COVID-19 comparative period for W1 *n* = 6,977)

	Wave	*n*(%)	Odds Ratio	95% CI	*p*-value
Any Respiratory intervention	1	3695 (45.37)	0.45	0.42–0.48	< 0.0001
	2	9243 (51.66)	1.29	1.22–1.36	< 0.0001
	3	12,228 (55.88)	0.19	1.14–1.23	< 0.0001
	4	8355 (54.82)	0.96	0.92-1.00	0.0430
	5	10,587 (57.31)	1.11	1.06–1.16	< 0.0001
	6	7124 (60.91)	1.16	1.11–1.22	< 0.0001
Mask 90% FiO_2_	1	2666 (32.74)	0.61	0.57–0.65	< 0.0001
	2	6740 (37.67)	1.24	1.18–1.31	< 0.0001
	3	8759 (40.03)	1.11	1.06–1.15	< 0.0001
	4	5202 (34.13)	0.78	0.74–0.81	< 0.0001
	5	6270 (33.94)	0.99	0.95–1.04	0.7105
	6	3986 (34.08)	1.01	0.96–1.06	0.8044
Nasal Cannula	1	459 (5.64)	1.20	1.04–1.39	0.0123
	2	1457 (8.14)	1.48	1.33–1.65	< 0.0001
	3	2405 (10.99)	1.39	1.30–1.49	< 0.0001
	4	2316 (15.20)	1.45	1.37–1.54	< 0.0001
	5	3058 (16.55)	1.11	1.04–1.17	0.0007
	6	2302 (19.68)	1.24	1.16–1.31	< 0.0001
Nebulized Bronchodilators	1	509 (6.25)	0.41	0.37–0.46	< 0.0001
	2	1009 (5.64)	0.90	0.80-1.00	0.0511
	3	1157 (5.29)	0.93	0.86–1.02	0.1245
	4	1123 (7.37)	1.43	1.31–1.55	< 0.0001
	5	1508 (8.16)	1.12	1.03–1.21	0.0068
	6	1222 (10.45)	1.31	1.21–1.42	< 0.0001

## Discussion

This study investigated prehospital respiratory interventions at a national level during the six waves of the COVID-19 pandemic. No such data were previously reported, and this study is as such the first of its kind. In keeping with how severe COVID-19 infections primarily affect older patients, only a small minority of responses were in children [24]. Total daily respiratory-related responses peaked in keeping with the surges in overall new confirmed COVID-19 cases in Israel [30]. This phenomenon is in contrast to that seen in a study of EMS responses in New York City, which demonstrated a surge of respiratory and cardiovascular-related responses early in the pandemic, followed by a decline in responses to pre-pandemic levels over time [31]. Similarly, a study from France found a 121% increase in respiratory-related responses from March to May 2020, compared to the pre-pandemic period, a more dramatic change than demonstrated in this study [32]. This may be a result of Israel’s introduction of far-reaching social restrictions at an early stage in the pandemic [28]. From mid-March 2020 to the end of April 2020, a restriction was imposed, including limitations on leaving home. Likely, this accounted for the sharp decrease in ambulance responses during W1. Subsequently, Israel went through a series of six periods of social restrictions during 2020 and 2021, before discontinuing restrictions in January 2022 [33]. Further, in December 2020, corresponding to the beginning of W3, Israel commenced its national immunization program, reaching a coverage of 116 COVID-19 doses per 100 people and outpacing other nations in the Organization for Economic Co-operation and Development (OECD) [11, 34]. A booster campaign was launched in July 2021, initially for the elderly, five months after prior vaccination [35]. This may account for the spike in positive cases during W5 not being reflected in the number of respiratory-related EMS responses, as the vaccines have been shown to offer protection against severe disease [12, 36]. 

As of the start of W1, no prehospital intubations were conducted. In the context of COVID-19, intubation is complicated by its highly aerosolizing potential; thus, patient needs had to be weighed against staff safety concerns. At the onset of the pandemic, guidelines were reliant on preliminary and limited data. For example, a review from 2020 on the recommendations for prehospital airway management from Illinois did not recommend against prehospital intubation but emphasized the need for high first-attempt success to avoid aerosolization as well as the use of video laryngoscopy. Similarly, consensus guidelines documents from China and the UK published in the early stages of the pandemic identify endotracheal intubation as a high-risk procedure for staff, even in the hospital setting, which ideally should take place in a negative-pressure room and be performed by the most experienced staff member to maximize the chance of success at the first attempt [37, 38]. While MDA paramedics have the mandate to perform endotracheal intubation and insert LMAs in the prehospital setting, during the pandemic, MDA advised against this through its own internal guidelines, resulting in no prehospital intubations or LMA insertions having been performed in our study. Given that Israel is a small country with an area similar to New Jersey and with a high density of medical facilities, transportation times to hospitals are generally shorter than in other countries. This allows for intubation to be deferred until the procedure could be performed in the more controlled environment of a hospital. Indeed, the limited clinical need for prehospital intubation in the context of short prehospital transportation distances has been demonstrated in a study of 256 patients with suspected COVID-19-related acute respiratory distress syndrome in Paris, France. This study demonstrated that only 7% of patients underwent prehospital intubation in this context, although comparisons of paramedical practices between countries are precarious due to differing scopes of practice and the underlying health status of the population [39]. 

Inter-wave analysis demonstrated that the odds for performing any intervention decreased sharply in the first wave but then gradually increased throughout the pandemic. This observation may be explained by the expanded vaccination coverage among staff over time, who may have been more comfortable performing interventions at later stages of the pandemic, as well as the health-seeking behavior of the patient population. The use of mask-based 90% FiO_2_ decreased in W1, but then increased during W2-3, after which the use decreased and plateaued towards the end of the pandemic. The initial drop in the use of high-concentration oxygen therapy may reflect staff concerns over its aerosol-generating potential in the confined space of an ambulance when no vaccine was available. The subsequent increased use of high-concentration oxygen in later waves may be due to providers feeling more comfortable performing the procedure as vaccine coverage expanded [40]. In contrast, the odds of nasal cannula use increased steadily throughout the pandemic, with the inter-wave ORs being continually above 1 throughout W1-6. The observation that over time, nasal cannula was sufficient to maintain blood oxygenation and in part replaced the use of high-concentration oxygen, despite escalating overall numbers of responses, may support the hypothesis that the increased transmissibility and decreased pathogenicity of new strains of COVID-19 were reflected in the prehospital setting. While the use of nebulized bronchodilators was limited in the early phases of the pandemic with a decrease throughout the first half of the pandemic, its utilization steadily increased during W4-6. Similar to high-concentration oxygen therapy, the aerosol-generating nature of nebulized bronchodilators may have contributed to the initial drop in its use, and subsequent increase, as vaccination coverage among health care providers expanded [40] Indeed, its increased use may also have contributed to the decreased need for oxygen therapy among patients. Further, with restrictions being lifted, there may have been increased transmission of other respiratory pathogens. Rhinoviruses, enteroviruses and *Chlamydophilia pneumoniae* are pathogens that are known to account for more infectious exacerbations of asthma than coronaviruses [41–43]. Similarly, rhinoviruses, *Hemophilus influenzae*,* Streptococcus pneumoniae* and *Moxarella catarrhalis* predominate in exacerbations of chronic obstructive pulmonary disease (COPD) [44–46]. Thus, increased demand for bronchodilators can reflect an increased incidence of infectious exacerbations of asthma and COPD secondary to the above pathogens.

The chief limitation of this study was the fact that the diagnosis of COVID-19 was not possible to make in the prehospital setting, nor was it possible to retroactively match EMS responses to in-hospital records. The effect of emerging strains on prehospital respiratory management needs therefore cannot be directly investigated. This may be especially true towards the latter waves of the pandemic when restrictions were eased, which affected the transmission of other respiratory pathogens. However, since social restrictions decrease the prevalence of non-COVID-19 pathogens as well, during the early phases of the pandemic in particular, respiratory infections severe enough to warrant ambulance transport are likely have been due to true COVID-19 infection [47–49]. Further, the fact that peaks in respiratory EMS responses follow the peaks in new confirmed COVID-19 cases indicate that most of these were in fact COVID-19 infections. Other limitations also affect the conclusions that can be drawn regarding the effect of the different strains. First, since Israel was the first nation to initiate a vaccination program, and COVID-19 vaccines have been demonstrated to decrease disease severity, expanded vaccination coverage may have contributed to the decreased need for oxygen therapy in the prehospital setting over time [10]. Second, eased social restrictions have likely contributed to the increase in the volume of responses later throughout the pandemic as society opened up.

## Conclusion

This study has described the population-wide needs of prehospital respiratory interventions during the COVID-19 pandemic in Israel and the trends throughout the 6 waves. While the reasons for prehospital management needs are multifactorial, including new strains of COVID-19, vaccine coverage, and changing practices, this study has demonstrated how needs have changed over time at the point of care. The analysis demonstrated an increased volume of responses over time, and a decreased need for prehospital high-concentration oxygen to maintain blood oxygen saturation which, in part, may be explained by the increased transmissibility and decreased pathogenicity of new strains. Additionally, an increased use of nebulized bronchodilators was demonstrated in the later waves, possibly owing to expanded vaccine coverage among providers and the re-emergence of non-COVID-19 pathogens as COVID-19 related restrictions were eased.

## Data Availability

Data available from Magen David Adom in Israel upon resonable request.
