# Correction: Prehospital respiratory interventions during six waves of COVID-19: results from Israel’s Emergency Medical Services system

**DOI:** 10.1186/s12873-025-01374-x

**Published:** 2025-11-04

**Authors:** Maximilian P. Nerlander, Evan Avraham Alpert, Roman Sonkin, Ziv Dadon, Ari M. Lipsky, Eli Jaffe

**Affiliations:** 1 Center for Disaster Medicine and Traumatology, Linköping University, Linköping, Sweden; 2 Community Division, Magen David Adom, Or Yehuda, Israel; 3 Faculty of Medicine, Hebrew University of Jerusalem, Jerusalem, Israel; 4 Department of Emergency Medicine, Hadassah Medical Center, Ein Kerem, Jerusalem, Israel; 5 Department of Internal Medicine, Soroka Medical Center, Beer-Sheva, Israel; 6 Jesselson Integrated Heart Center, Shaare Zedek Medical Center, Eisenberg R&D authority, Jerusalem, Israel; 7 Department of Emergency Medicine, HaEmek Medical Center, Afula, Israel; 8 Rappaport Faculty of Medicine, Technion-Israel Institute of Technology, Haifa, Israel; 9 Faculty of Health Sciences, Ben-Gurion University of the Negev, Beer-Sheva, Israel

The published manuscript states that no intubations were performed by Magen David Adom (MDA) paramedics from the start of the COVID-19 pandemic due to a recommendation not to do so. However, it has come to the attention of the authors that intubations were indeed performed. The reason for our data not reflecting this is because MDA data collection for intubations and other advanced airway interventions required the paramedic to indicate that the patient was ventilated and to separately tick a box indicating oxygen delivery through the advanced airway (either endotracheal tube [ET] or laryngeal mask airway [LMA]). The data collection practice changed at the very beginning of COVID-19, eliminating the need to tick oxygenation via advanced airway (because by directive all MDA patients who are ventilated by ET or LMA receive oxygen). Because the oxygenation variable was used to identify potential advanced airway interventions, the number of patients ventilated via an advanced airway during the six waves of COVID-19 in our analysis was incorrectly stated as 0.

Upon re-analysis, there were a total of 3,267 patients ventilated using advanced airways in the population of 141,027 transported by MDA during the six waves of COVID-19 (2.3%). The correct number and percentage of patients ventilated through advanced airways by wave were added to Table 2. While we had chosen a threshold of 3% for inclusion of procedures in the inter-wave analysis and the advanced airways interventions were below this threshold, given their importance we added them to the odds ratios (ORs) displayed in Table 3.

In our original analysis, patients with an advanced airway (whether ET or LMA) were included appropriately in the denominator. Thus, no other results are affected by this correction.

The specific sections and sentences of the published study that are affected are as follows:

## Abstract

The **Abstract Results** section should now read as follows (the 2nd sentence was replaced):

The study included 141,027 responses. The use of advanced airways decreased between the reference period and W1 (OR 0.56, *p* = <0.0001 [W1]), fluctuated during W2 and W3 (OR 1.54 *p* = <0.0001 [W2]; OR 0.85, *p* = 0.02 [W3]), remained steady during W4 and W5 (OR 1.09, *p* = 0.2611 [W4]; OR 1.12, *p* = 0.13 [W5]) and then increased in W6 (OR 1.18, *p* = 0.03 [W6]). The use of mask-based 90% FiO2 decreased from the pre-COVID-19 period to W1 (OR 0.61, *p* < 0.0001), increased during waves 2–3 (OR 1.24, *p* < 0.0001 [W2]; OR 1.11, *p* < 0.0001 [W3]), and plateaued throughout W5 and W6 (OR 0.99, *p* = 0.71 [W5]; OR 1.01, *p* = 0.8 [W6]). The use of nasal cannula increased throughout the six waves (OR 1.2 [W1]; OR 1.48 [W2]; 1.39 [W3]; OR 1.45 [W4]; 1.11 [W5]; 1.24 [W6], *p* < 0.05). The use of nebulized bronchodilators decreased from the pre-pandemic period to W1 (OR 0.41, *p* < 0.0001). From W3 to W6, the use increased significantly for each wave (OR 1.43 [W4]; OR 1.12 [W5]; 1.31 [W6], *p* < 0.05).

## Manuscript

In the **Manuscripts Results**, third paragraph, the following text should replace the second sentence: “Interventions accounting for less than 3% of total interventions during COVID-19 were omitted from the comparative analysis. However, an exception was made for advanced airways due to their clinical importance.”

Table 2 should be replaced with the accompanying Table 2. The new table replaces the two last rows with a new row, includes more explanatory text in the title, and corrects a rounding error (percentage of any intervention, total during COVID-19, is 57.1, not 57.0).

Table 3 should be replaced with the accompanying Table 3. The new table adds a new row section and corrects a typo (p-value of nasal cannula, W1, should be 0.0125, not 0.0123).

## Discussion

The second paragraph of the discussion should be replaced with the following paragraph (only the opening sentence and the sentence beginning “While MDA paramedics” in the middle of the paragraph were modified from the original):

As of the start of W1, the use of ET tubes and LMAs decreased, and it was only toward the end of the pandemic, in W6, that their use returned to the level of use seen in the pre-pandemic reference period. In the context of COVID-19, intubation is complicated by its highly aerosolizing potential; thus, patient needs had to be weighed against staff safety concerns. At the onset of the pandemic, guidelines were reliant on preliminary and limited data. For example, a review from 2020 on the recommendations for prehospital airway management from Illinois did not recommend against prehospital intubation but emphasized the need for high first-attempt success to avoid aerosolization as well as the use of video laryngoscopy. Similarly, consensus guidelines documents from China and the UK published in the early stages of the pandemic identified endotracheal intubation as a high-risk procedure for staff, even in the hospital setting, which ideally should take place in a negative-pressure room and be performed by the most experienced staff member to maximize the chance of success at the first attempt [37, 38]. While MDA paramedics had the mandate to perform endotracheal intubation and insert LMAs in the prehospital setting, during the pandemic, MDA via a protocol distributed to paramedics advised extreme caution if there was a decision to intubate in the case of COVID-19. This resulted in the limited use of prehospital intubations or LMA insertions as demonstrated in our study. Given that Israel is a small country with an area similar to New Jersey and with a high density of medical facilities, transportation times to hospitals are generally shorter than in other countries. This allowed for intubation to be deferred until the procedure could be performed in the more controlled environment of a hospital. Indeed, the limited clinical need for prehospital intubation in the context of short prehospital transportation distances has been demonstrated in a study of 256 patients with suspected COVID-19-related acute respiratory distress syndrome in Paris, France. This study demonstrated that only 7% of patients underwent prehospital intubation in this context, although comparisons of paramedical practices between countries are precarious due to differing scopes of practice and the underlying health status of the population [39].

The authors apologize for these errors.

**Table 2:** Characteristics of the study population (*n* = 141,027; pre-COVID-19 reference period *n* = 6,977. Percentages in “Reference” column to “W6” denote proportions with the entire study population as denominator, percentages in “Total during COVID-19” column only includes the COVID-19 period as denominator)

	Reference	W1	W2	W3	W4	W5	W6	Total during COVID-19
Dominant Stain of COVID-19	N/A	Original strain	Original strain	Alpha B.1.1.7	Delta	Omicron	Omicron	N/A
Age (Median, [IQR])	76.1 [62–85.3]	66.2 [44.1–81.3]	68.1 [48.8–82]	70 [50.1–83]	68.7 [47.3–82.4]	74.2 [58.1–85.2]	75.1 [61.1–85.2]	71.4 [52.7–84.0]
Male (n, %)	3,383 (48.49)	4,163 (51.12)	8,913 (49.81)	10,675 (48.79)	7,380 (48.43)	8,760 (47.42)	5,728 (48.97)	72,141 (48.80)
Female (n, %)	3,594 (51.51%)	3,981 (48.88)	8,980 (50.19)	11,206 (51.21)	7,860 (51.57)	9,713 (52.58)	5,968 (51.03)	75,863 (51.20)
Interventions								
Any (n, %)	4,520 (5.32)	3,695 (4.35)	9,243 (10.88)	12,228 (14.39)	8,355 (9.83)	10,587 (12.46)	7,124 (8.38)	80,452 (57.1)
Mask 90% FiO2 (n, %)	3,107 (5.68)	2,666 (4.88)	6,740 (12.33)	8,759 (16.02)	5,202 (9.51)	6,270 (11.47)	3,986 (7.29)	51,578 (36.6)
Nasal cannula (n, %)	330 (1.73)	459 (2.40)	1,457 (7.63)	2,405 (12.60)	2,316 (12.13)	3,058 (16.02)	2,302 (12.06)	18,758 (13.3)
Nebulized Bronchodilators (n, %)	971 (7.58)	509 (3.98)	1,009 (7.88)	1,157 (9.04)	1,123 (8.77)	1,508 (11.78)	1,222 (9.54)	11,834 (8.4)
Mask 60% FiO2 (n, %)	367 (9.16)	210 (5.24)	465 (11.61)	555 (13.85)	313 (7.81)	412 (10.28)	237 (5.92)	3,639 (2.6)
Bag valve mask (n, %)	132 (3.80)	123 (3.54)	447 (12.88)	457 (13.17)	339 (9.77)	430 (12.39)	327 (9.42)	3,338 (2.4)
Advanced airway: Endotracheal Tube Intubation (ETT) or Laryngeal Mask Airway (LMA) (n, %)	187 (5.41)	123 (3.56)	412 (11.93)	430 (12.45)	325 (9.41)	440 (12.74)	326 (9.44)	3267 (2.32)
Continuous Positive Airway Pressure (CPAP) (n, %)	148 (8.98)	9 (0.55)	91 (5.52)	177 (10.74)	145 (8.80)	230 (13.96)	169 (10.25)	1,500 (1.1)

**Table 3:** Odds ratios for respiratory interventions across the six Waves. Each wave was compared to the previous one, W1 was compared to a pre-COVID-19 reference Period. (*n* = 141,027; pre-COVID-19 reference period for W1 *n* = 6,977. Percentages denote proportion of each wave)

	Wave	*n* (%)	Odds Ratio	95% CI	*p*-value
Any Respiratory intervention	1	3,695 (45.37)	0.45	0.42–0.48	< 0.0001
	2	9,243 (51.66)	1.29	1.22–1.36	< 0.0001
	3	12,228 (55.88)	1.19	1.14–1.23	< 0.0001
	4	8,355 (54.82)	0.96	0.92-1.00	0.0430
	5	10,587 (57.31)	1.11	1.06–1.16	< 0.0001
	6	7,124 (60.91)	1.16	1.11–1.22	< 0.0001
Mask 90% FiO_2_	1	2,666 (32.74)	0.61	0.57–0.65	< 0.0001
	2	6,740 (37.67)	1.24	1.18–1.31	< 0.0001
	3	8,759 (40.03)	1.11	1.06–1.15	< 0.0001
	4	5,202 (34.13)	0.78	0.74–0.81	< 0.0001
	5	6,270 (33.94)	0.99	0.95–1.04	0.7105
	6	3,986 (34.08)	1.01	0.96–1.06	0.8044
Nasal Cannula	1	459 (5.64)	1.20	1.04–1.39	0.0125
	2	1,457 (8.14)	1.48	1.33–1.65	< 0.0001
	3	2,405 (10.99)	1.39	1.30–1.49	< 0.0001
	4	2,316 (15.20)	1.45	1.37–1.54	< 0.0001
	5	3,058 (16.55)	1.11	1.04–1.17	0.0007
	6	2,302 (19.68)	1.24	1.16–1.31	< 0.0001
Nebulized Bronchodilators	1	509 (6.25)	0.41	0.37–0.46	< 0.0001
	2	1,009 (5.64)	0.90	0.80-1.00	0.0511
	3	1,157 (5.29)	0.93	0.86–1.02	0.1245
	4	1,123 (7.37)	1.43	1.31–1.55	< 0.0001
	5	1,508 (8.16)	1.12	1.03–1.21	0.0068
	6	1,222 (10.45)	1.31	1.21–1.42	< 0.0001
Advanced Airway: Endotracheal Tube Intubation (ETT) or Laryngeal Mask Airway (LMA)	1	123 (1.51)	0.56	0.44–0.70	< 0.0001
	2	412 (2.30)	1.54	1.25–1.88	< 0.0001
	3	430 (1.97)	0.85	0.74–0.98	0.02
	4	325 (2.13)	1.09	0.94–1.26	0.2611
	5	440 (2.38)	1.12	0.97–1.30	0.13
	6	326 (2.79)	1.18	1.02–1.36	0.03
